# Green and Sustainable Valorization of Bioactive Phenolic Compounds from *Pinus* By-Products

**DOI:** 10.3390/molecules25122931

**Published:** 2020-06-25

**Authors:** Pedro Ferreira-Santos, Elisa Zanuso, Zlatina Genisheva, Cristina M. R. Rocha, José A. Teixeira

**Affiliations:** CEB—Centre of Biological Engineering, University of Minho, Campus de Gualtar, 4710-057 Braga, Portugal; pedrosantos@ceb.uminho.pt (P.F.-S.); elisa.zanuso@ceb.uminho.pt (E.Z.); zlatina@ceb.uminho.pt (Z.G.); cmrocha@ceb.uminho.pt (C.M.R.R.)

**Keywords:** pine, by-products, biorefinery, green process, polyphenols, biological activity, traditional applications, high value-added products

## Abstract

In Europe, pine forests are one of the most extended forests formations, making pine residues and by-products an important source of compounds with high industrial interest as well as for bioenergy production. Moreover, the valorization of lumber industry residues is desirable from a circular economy perspective. Different extraction methods and solvents have been used, resulting in extracts with different constituents and consequently with different bioactivities. Recently, emerging and green technologies as ultrasounds, microwaves, supercritical fluids, pressurized liquids, and electric fields have appeared as promising tools for bioactive compounds extraction in alignment with the Green Chemistry principles. Pine extracts have attracted the researchers’ attention because of the positive bioproperties, such as anti-inflammatory, antimicrobial, anti-neurodegenerative, antitumoral, cardioprotective, etc., and potential industrial applications as functional foods, food additives as preservatives, nutraceuticals, pharmaceuticals, and cosmetics. Phenolic compounds are responsible for many of these bioactivities. However, there is not much information in the literature about the individual phenolic compounds of extracts from the pine species. The present review is about the reutilization of residues and by-products from the pine species, using ecofriendly technologies to obtain added-value bioactive compounds for industrial applications.

## 1. Introduction

Agroforestry industries are an important part of the manufacturing industry, and their growth can help to achieve the objectives of European Union (EU) industrial policy, acting in different strategic areas, such as increasing energy efficiency, deploying renewable sources, circular economy, bioeconomy, and natural carbon sinks [1,2]. Moreover, the development of these industries should also be in line with the 17 sustainable development goals by 2030 dictated by the United Nations; in particular, the agroindustry can directly impact on at least 4 of these goals related to the use of clean energy, industry innovation, responsible consumption, and climate action [3].

Nowadays, 5 billion tons of biomass residues from agroforestry and food industries are estimated worldwide and represent an emission for 3.3 billion tonnes of carbon dioxide each year [4,5]. In the EU, the total annual biowaste is estimated at around 100 million tonnes, generating a negative ecological impact [6].

One of the strategies for the reduction of generated environmental impact is the reuse of industrial biowastes to obtain new natural ingredients. Concomitantly, the growing interest in the development of effective/intensified processes and application of green technologies to obtain sustainable, ecological, safe and high-quality products has become a reality [7,8]. This idea is in close association with the principles governing the concept of green chemistry, which are mainly aimed at reducing wastes and promoting a more efficient use of energy and resources [9].

The decrease in the use of “non-recyclable” fossil derivatives and the increase in the use of biowastes and by-products is in the sights of the EU and the world, contributing to the reduction of the negative impact of processes in the environment and the fight against climate changes [2]. In this sense, the use of different plant by-products as sources of materials, biofuels, energy, and bioactive compounds has come to be explored following the concept of biorefinery, contributing to a circular economy [6].

The present review focuses on the appreciation of different green extraction strategies related to the recovery of high added-value compounds (such as polyphenols) from pine by-products, their potential bioactivities, and possible industrial applications. 

## 2. Biorefinery and Lignocellulosic By-Products

**Figure 1:**
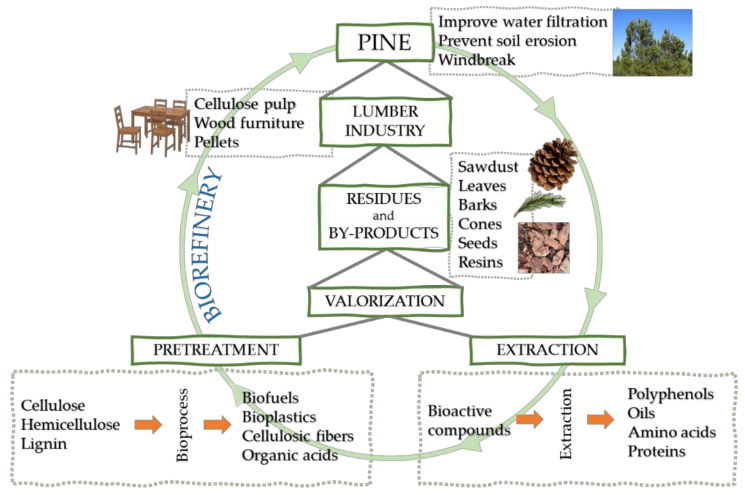
Pine valorization under biorefinery concept.

Resources depletion, waste accumulation, and climate change are a combination of forces driving the need for the sustainable practices we are facing nowadays. Additionally, urbanization and population growth are causing the global energy demand to be in continuous rise. With the energy demand increasing, the necessity of detachment from fossil fuels and the transition to renewable resources is mandatory to reduce the environmental problems. Energy resources such as biomass, wind, and solar energy can meet the energy requirements if large-scale technologies are well developed [10]. In this sense, biorefinery is analogous to fossil fuel refinery. The biorefinery term dates back to 1980. Since then, several definitions have been considered. These definitions are based on the type of feedstock used, type of processes, and type of products obtained [11]. In general, the biorefinery concept is the synergy of technologies that convert biomass into their building blocks to produce a variety of biofuels, chemicals, and high added-value compounds. Hereof, the development of a sustainable process involves not only the use of biomass but also implies reducing the use of harmful chemicals, transition to greener processes, efficient use of energy, and elimination of wastes (Figure 1) [9]. 

Ethanol is a well-known biofuel product of the second generation biorefinery. Second generation or lignocellulosic biorefinery usually begins with the pretreatment of the biomass in order to increase the digestibility of cellulose, to solubilize the hemicellulose, and to relocate the lignin [12]. Pretreatments can be mechanical, chemical, physicochemical, or biological [13]. Afterward, to liberate the fermentable sugars (i.e., monosaccharide units) from the pretreated biomass, acid or enzymatic hydrolysis processes are applied [14]. Then, the fermentation of the obtained sugars to obtain ethanol can be carried out using microorganisms as yeast or bacteria [15,16], where, in an integrated biorefinery, ethanol can also be considered as a precursor for chemicals, hydrocarbon fuels, and aromatic compounds [17]. Hence, the research and development of sustainable processes are growing not only for biofuels but also for the high added-value compounds than can allow an economically viable process (Figure 1).

Currently, lignocellulosic biomass has been largely studied as a potential substrate in fermentation processes, and mainly for biofuel production. Nevertheless, innovative and new emerging technologies are being studied to increase the obtainment of high-value compounds of interest, particularly bioactive ones. The recovery of these of high-value compounds is linked to the biorefinery concept and the green chemistry principles. Lignocellulosic biomass (in which pine by-products may be included) is mostly considered as a residue from crops as straw, sugarcane bagasse, corn stover, and wood waste. Cellulose is the major component of the lignocellulosic materials, followed by hemicellulose and lignin. Cellulose is the world’s most abundant biopolymer made up of glucose units. Applications of cellulose extracted from lignocellulosics include the manufacture of cellulosic fiber and nanocrystalline cellulose in a wide range of industries such as automotive, textile, and medicine due to the strength on its structure, availability, modifiable surface, renewability, and low cost [18,19]. Hemicellulose is the second most abundant polysaccharide in lignocellulosic biomass mainly composed by monomeric units as xylose, mannose, arabinose, glucose, galactose, and acids such as uranic acid [20]. Hemicellulose and specific target products from hemicellulose are used in a variety of areas as food, medicine, and chemicals due to the biocompatibility and bioactivity properties they show [20]. Pine sawdust has been used to produce levulinic, formic, and acetic acid and furfural form hemicellulose extracted by steam explosion [21]. In addition, pinewood (*Pinus eldarica)* pretreated with dilute sodium hydroxide was used to produce ethanol from the pretreated solid where the solubilized hemicellulose fraction was used to produce biogas [22]. The third main component of lignocellulosic materials is lignin, which is an amorphous phenolic polymer that provides mechanical strength and rigidity to plants [23]. In the biorefinery process, lignin cannot be used as a substrate for fermentation as it contains no sugars. Therefore, a wide area of research is on lignin valorization. Lignin is mainly used to generate heat and electricity due to the high heating value although other applications are possible, including the use as a precursor for carbon fiber synthesis, resins, and low molecular weight aromatic and phenolic compounds [24,25]. Nowadays, the modern polymer industry from natural sources of aromatic compounds is limited due to the high prices of the final product. Here, lignin plays an important role, since phenolic compounds can be obtained from lignin deconstruction [26]. 

Historically, wood has been a major energy source for human beings. Forest biomass is the most abundant feedstock on earth, representing 89.3% of the total biomass [27]. In Europe, forest area is one of the most important renewable resources, representing near 5% of the world’s forest and covering 43% of its land, comprising close to 182 million hectares of forest. Forest is also considered as a resource for improving life quality and job generation [28]. Wood biomass can be densified into solid fuels, as pellets, or converted into heat, electricity, biofuels, and other bioproducts through a variety of chemical, thermochemical, and biochemical processes [29]. On the other hand, the lumber industry generates a considerable amount of waste that includes leaves, barks, sawdust, chips, cones, resins, and branches. These residues are not usually well valued and are thrown out, burned, or used for animal bedding, although they can be a profitable source of high added-value compounds [30]. Therein, wood biomass residues increase their overall value due to the metabolites that are present in lower abundance compared with cellulose, hemicellulose, and lignin. These extractive compounds combine alkaloids, waxes, phenolics, pectins, resins, and essential oils [31], and they are of great importance considering the wide industry applications, more precisely, in food and pharmaceutical industries due to the antioxidant, antimicrobial, anti-inflammatory, and antitumoral effects they show [32].

## 3. Pine as Feedstocks

This work is mainly focused on the valorization of the *Pinus* species (and its by-products), which are evergreen trees of resinous conifers group from the *Pinaceae* family. In the EU, there are more than 14 different species, representing one of the largest forest occupations. Table 1 describes the main *Pinus* species distributed in the European countries according to the “European forest genetic resources program (EUFORGEN)” [33]. In Portugal, pine forests are the third forest formation after eucalyptus and cork oaks, with an area of approximately 1 million hectares, representing an important part of the total forest, about 23% [34].

The chemical composition of pine and its constituents (wood, bark, leaves, cones, seeds, and resin) varies depending on the *Pinus* tree and also on many other factors, such as genotypic, ecological, and seasonal, among others [35]. The methodology used for the determination of chemical composition of plant resources is also a factor to consider, since different methods lead to different results [36]. 

**Figure 2:**
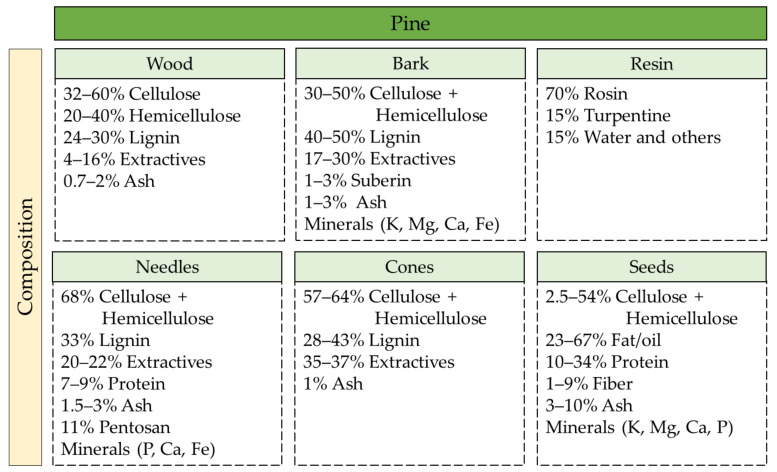
General chemical/nutritional composition of pine by-products.

The general chemical/nutritional composition of pine by-products has been described by several authors (wood [37,38,39,40,41,42], bark [43,44,45,46,47], needles [48,49], cones [41,50], seeds (nuts) [51,52,53], and resin or oleoresin [54,55]) and is summarized in Figure 2. 

### Pine Applications

At present, agroforestry residues and by-products are mainly used as combustion feedstock for biofuels production [56]. The most important biomasses are obtained from lumber industry (bark and sawdust) or forest activities, the residues from farms and agro-business, the organic fraction of municipal solid wastes, and the plants deliberately grown for energetic purposes. In this sense, it is important to reduce and give a “second life” to these residues, moving to “zero waste”.

The *Pinus* plant is very important economically, as it is considered good feedstock for the bioeconomy (Figure 1) [57]. In its natural environment, it has an important protective function, such as improving water infiltration, preventing soil erosion on dry slopes, and serving as a windbreak [33]. Trees are also used as ornamental plants in urban and industrial contexts. Other uses include Christmas trees and fuelwood.

Interestingly, in a study by Ehn and co-workers [58], it has been found that pine forest aroma (for its content in volatile compounds, terpenes) can limit climate change, preventing the global warming.

The main industrial activities are related to the usage of pine wood and wooden products, including sawmills, wood panels, cellulose pulp and paper production, wood fuels, carpentry, packing, and wood furniture [33]. These feedstock, their components, and their by-products are considered a good source for wood biorefineries, transforming the lignocellulosic fractions into biofuels, chemical products, and composite materials, as previously mentioned [57].

Pine bark, the by-product obtained in larger quantities that is produced when wood is transformed, is almost exclusively used as fuel, being also subjected to composting to filling substrate in nurseries, utilized for cover in public gardens, or simply thrown away on landscapes [44,59]. Nowadays, this by-product has been used as low-cost and green alternatives waste-based biosorbents for the removal of a wide range of water pollutants [60].

The pine leaves (needles) are normally used in agriculture to enrich the soil, and the seeds are used for human consumption because they are highly nutritious and much appreciated by consumers in cooked/prepared dishes (food industry) or simply as edible pine nuts. The resins, a product resulting from the exploitation of these species, are more regularly used as a sealant, glue, varnish, and also as a solvent and paint thinner (turpentine oil) [55,57,61].

In addition to the “traditional” uses of these by-products, it is important to take advantage of these bio-resources to create high value-added products.

Currently, the agroforestry by-products have been increasingly exploited to isolate biocompounds with high industrial interest. Studies using natural matrices as a potential source of bioactive compounds have been published in recent decades [7,62,63,64,65,66,67]. For instance, in a new review paper [68], the authors report that in addition to fruits and vegetables, tree barks are rich in phenolic compounds with excellent biological properties (such as antioxidant, immunostimulatory, anticancer, antibacterial, anti-inflammatory, antimutagenic, etc.) and may used to obtain functional ingredients.

Pine bark is one of the most sought after sources of antioxidant biocompounds of natural origin. The extracts obtained from this by-product are mostly composed of phenolic compounds with high biological activity [46,59,62,69,70,71,72]. Nowadays, there are numerous studies reporting the applicability of pine bioactive extracts in various areas, such as health care, food, agrochemical, and others [73,74,75]. One of the most promising applications for these extracts is in the preservation and enrichment of foods, thus replacing synthetic antioxidants, as well as a nutraceutical, cosmeceutical, or pharmaceutical. 

The pine wood/sawdust extractives, rich in phenolic antioxidant compounds, have a potential for food and pharmaceutical applications, such as preservatives or nutraceuticals [40,76]. 

Pine tars, a by-product of pine wood and bark, are known to contain tricyclic diterpenoid resin acids, tricyclic diterpene hydrocarbons, alkylphenanthrenes, and fatty acids. This water-resistant by-product has a wide range of applications, for example, as a multipurpose adhesive, sealant, and in medicine [77,78].

Knowing the chemical composition and physicochemical properties, pine seeds or nuts appear to have a positive effect on human health [52,53]. The seed lipids, rich in linoleic acid, have a beneficial effect on blood pressure and cholesterol. The fatty acid composition and the relatively high polyphenol content present high protection against oxidative stress. In this sense, pine seeds can potentially be used in the food industry and other non-food industries, such as pharmaceutical and cosmetics [79,80,81].

Oleoresins are widely used in the synthesis of perfumed compounds for cosmetics, essences as additives for food and beverages, food protection (antimicrobial), bioinsecticides (high repellent activity), tapping green chemicals, biofuels, and carbon sequestration from multipurpose trees [54,55,57,61].

Interestingly, this search for functional extracts, new natural molecules, and the creation of new high value-added products has increased the use/study of agroforestry by-products and residues, including pine bark, sawdust, leaves, seeds, and resin. This makes it possible to potentially bring these “wastes” back to the market.

## 4. Extraction Processes for Phenolic Compounds Recovery

The recovery of bioactive and functional purified biomolecules or extracts from plant materials is an important step to enable the reuse of natural resources for subsequent application in pharmaceutical and cosmetic products, food enrichment and preservatives, dietary supplements, and nutraceuticals. 

The extraction process of natural extracts depends on several factors, including the applied extraction technique, the parameters associated with the technique (such as temperature, time, and the extraction solvent), and the raw materials composition [63]. It is known that the phenolic compounds are metabolites present in the cell vacuoles [82]. Therefore, it is also important to promote the opening of pores or even the rupture of the cell wall to facilitate the release of the compounds into the extraction medium.

**Figure 3:**
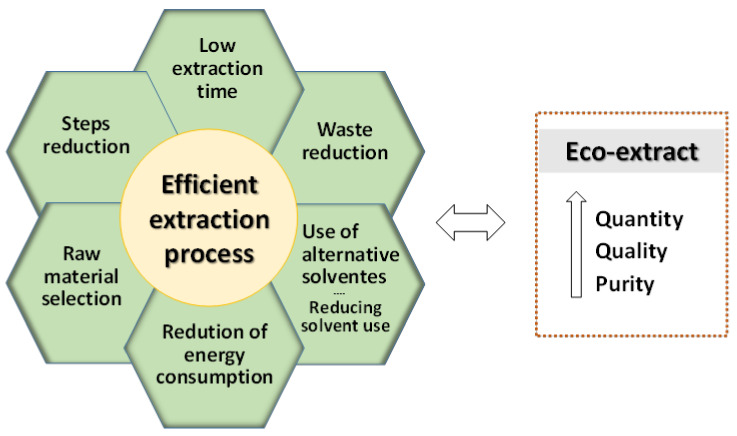
Principles of efficient process for obtaining natural extracts. Adapted from Chemat et al. [8].

In this sense, it is important to study all variables of the process in order to maximize the potential of the extraction method, developing a highly efficient process [8]. On the other hand, all variables in the process have to make it possible to obtain a safe and high quality final product (eco-extract), in addition to maximizing the extraction of the compounds of interest. Figure 3 illustrates the main principles of an efficient extraction process, following the concept of green extraction. 

### 4.1. Extraction Solvents

The reduction use of hazardous solvents is also considered one of the priorities of the EU policy for the 2010 to 2050 period [7]. Nowadays, extraction using conventional organic solvents is the most commonly used procedure to prepare extracts from plant materials due to their ease of use, efficiency, and wide applicability. The efficiency of the extraction methods depends on the choice of the solvent, since solvents with different polarities are needed for the isolation of compounds with different chemical constitution. In addition, it is difficult to define a single method for the efficient extraction of all compounds, since the polarities of the molecules to be extracted vary [63]. 

A suitable solvent has to be able to obtain safe and high-quality extracts and to preserve the biological effects of the extracted compounds without exhibiting toxicity when consumed. Furthermore, it should be recyclable and reusable, preventing negative environmental effects. Other parameters, such as flammability, explosiveness, volatility, mass transfer, and (in)ability to dissociate the complex extract should be considered [65]. The extraction yield depends not only on the solvent used but also on several other factors such as sample/solvent ratio, temperature, extraction time, stirring, and raw material composition [83].

Conventional solvents from “non-natural”/petroleum resources, such as methanol, ethanol, acetone, ethyl acetate, dichloromethane, hexane, etc. and their aqueous solutions have been used for the extraction of bioactive compounds from plant materials. Several studies have been done demonstrating the importance of these solvents in the recovery of natural molecules and active extracts from different plants and by-products [65,84,85,86], including the lignocellulosic by-products [87]. Researchers also studied the influence of these different solvents in obtaining antioxidant phenolic compounds from pine by-products, and depending on the solvent used, the extracted fraction (extract composition) is different [59,85,88]. For example, in a work by Venkatesan and collaborators [85], the impact of different extraction solvents (such as ethanol, methanol, isopropanol, acetonitrile, and acetone) was analyzed to obtain phenolic extracts with antioxidant activity from *Pinus densiflora* bark. Their results showed that low concentrations of ethanol and acetonitrile are favorable for the extraction of phenolics with high antioxidant activity. In another study, using *Pinus niruri*, methanol was more efficient than other solvents such as ethanol, hexane, and ethyl acetate, showing an enhanced extraction rate of phenolic and flavonoid compounds with higher biological activities [88].

It is known that water is an efficient solvent for the extraction of various compounds, due to its properties and thanks to the fact that water is easily available, safe, non-toxic, non-flammable, and environmentally friendly [8]. In this sense, it is considered the cleanest/greenest solvent (apart from the use of no solvent, which is the greenest), according to the principles of green chemistry [64,89]. However, it is not suitable for the extraction of less polar substances.

Other possible environmental friendly solvents’ option is to replace petroleum-based solvents by “bio-solvents”. For instance, “bioethanol” can be produced from bioresources, by fermentation. This second-generation solvent could be made cost-competitive by the development of biorefinery-based processes for the integral use of lignocellulosic biomass, substituting ethanol obtained from petroleum derivatives [90].

As alternatives to conventional solvents, the use of green solvents such as ionic liquids (ILs) and natural deep eutectic solvents (NADES) is emerging, in order to make the extraction process eco-friendly and more effective [91]. In general, ILs and NADES are derived from cheap, abundant, low toxic, and biodegradable natural components [6,7]. NADES can be defined as “mixtures of pure naturally occurring compounds that present an eutectic point temperature below an ideal liquid mixture” [6,92]. ILs are liquid molten salts at temperatures below 100 °C composed by cations and organic or inorganic anions with exclusive and adjusted physicochemical properties [93].

However, a lack of information on the biological activity and toxicity of the obtained extracts limits the use and industrial applications of ILs and NADES [7], leading to these solvents not being regulated by the Federal Drug Administration (FDA) [94]. Furthermore, although they can be tuned for enhanced affinity toward the compound of interest, their separation from the final mixture may be hindered by the high boiling point characteristic of these solvents.

Murador and collaborators [95] summarize the main chemical constituents of these ILs and NADES and mention some works where they are applied in the extraction of antioxidant compounds, such as phenolic compounds and carotenoids, among others. Specifically, ILs and NADES have been applied to the phenolic compounds and other antioxidant compounds extraction from lignocellulosic biomass and agri-food wastes [4,6,93,96,97,98].

In the case of pine plants as feedstock, the process of extracting bioactive compounds with added value (such as phenolics) using these green solvents is not widely explored. In a recent study, ILs were combined with enzymes and microwave technology to promote cell wall disruption for the extraction of essential oil and procyanidins from pine cones of *Pinus koraiensis* [99]. However, there are no reports using ILs and NADES as alternative solvents for extraction of bioactive molecules of other parts of pine plant, despite the advantage they showed for obtaining functional compounds in other lignocellulosic residues.

### 4.2. Extraction Technologies

Conventional methods of extraction, such as the solid–liquid method, hydrodistillation, maceration, and Soxhlet require the use of large amounts of water or organic solvents, agitation, long extraction time, high temperatures, and energy consumption, as well as the generation of a considerable quantity of wastes [8,100,101].

The need for obtaining greener, sustainable, and viable processes has led scientists and industries to develop new processes in full correspondence with the green extraction concept [102,103]. In this context, the search for alternative extraction technologies with environmental and economic advantages, taking into account the characteristics of the final products has emerged [104]. As a result, techniques such as ultrasound-assisted extraction (UAE), microwave-assisted extraction (MAE), supercritical fluid extraction (SFE), pressurized liquid extraction (PLE), and ohmic heating (OH) electrotechnology have been developed, optimized, and applied to improve the extraction process of antioxidant phenolic compounds from plant resources, such as pine by-products (Table 2). 

In the following sub-sections, a brief introduction to these extraction technologies and some examples of their application in obtaining phenolic compounds from pine by-products will be presented.

#### 4.2.1. Ultrasound-Assisted Extraction

Ultrasound produces high-intensity sound waves (typically higher than 20 kHz) [104]. The operation mechanism of UAE is based in pressure variations that form microbubbles resulting in microturbulence and a high collision of particles. The collapse of microparticles caused by ultrasound waves can promote higher penetration of the solvent into the cellular material causing the cell walls disruption and increasing the release of intracellular compounds into the extraction medium [105,106]. UAE is an alternative technology with advantages compared to conventional techniques, since less processing time, low solvent usage, and lower extraction temperatures are required, preserving heat-sensitive compounds. In addition, it leads to an increase in extraction yield, requiring less energy in the process [106]. Due to its advantages, ultrasound technology is mentioned as an eco-friendly and cheap process and can be easily implemented to extract phenolic compounds from plants and plant by-products [76,107,108,109,110].

In the last decade, UAE has been used to obtain extracts rich in phenolic compounds from pine by-products. In a study of Liazid and co-workers [111], UAE was used to obtain phenolic extracts from seeds of two pine species (*Pinus maritima* and *Pinus d’Alpes*). The results of this work showed that the application of ultrasound waves, using water as a solvent at 75 °C during 20 min, doubled the recovery of phenolic compounds compared to a conventional maceration technique, increasing the antioxidant activity of these extracts. Using the bark of the *Pinus radiata* as a raw material, Aspé and collaborators [112] verified that the synergetic effect of ultrasounds (35 kHz/85 W) with acetone 70% (*v/v*) allows the formation of pores in the matrix cells, promoting the rapid rupture of the cell wall, facilitating the extraction of phenolic compounds, and drastically reducing the extraction time (from 180 min for conventional extraction in a water bath or Soxhlet, for 6 min when using UAE).

In another study, authors used ultrasound technology in combination with methanol 70% (*v/v*) as a solvent to extract phenolic compounds, such as flavonoids from leaves (needles) of four different pine species (*Pinus peuce*, *P. nigra*, *P. mugo* and *P. sylvestris*) [113]. UAE proved to be a potential tool for the sustainable recovery of phenolic compounds from pine leaves, without using temperature in the process. 

Recently, Meullemiestre and co-workers [76] reported that the UAE, in addition to increasing the extraction of phenolic compounds from maritime pine wood (sawdust waste) by 40% compared to conventional extraction techniques (solid–liquid), also allowed reducing the time of the process. On the other hand, they also reported that UAE is a scalable technique and can be applied industrially to obtain bio-functional extracts.

#### 4.2.2. Microwave Assisted Extraction

MAE is a heating process using electromagnetic waves of frequency between 300 MHz and 300 GHz that interact with samples to extract analytes from a matrix to a solvent. The microwave irradiation increases the internal pressure of the plant cells by heating the cells from the inside, leading to cell disruption and releasing the compounds of interest. Some of the advantages of MAE are a lower time of extraction compared with other extraction processes, the possibility of multiple extractions, low solvent volume, an attainment of high temperatures, and more effective, uniform and selective heating [107,114]. To have better extraction yields, it is important to consider the capability of the solvent to absorb microwaves, as it can be a drawback when the solvent lacks the capacity of energy absorption. Furthermore, the thickness of the sample to be heated may also be a drawback, particularly in the scalability of the extraction process, as the ability of microwaves to penetrate a sample is limited. Liazid et al. [111] studied the extraction of phenolic compounds from *Pinus pinaster* seeds using water as solvent and demonstrated that MAE produces extracts with great polyphenols content, since it can achieve high temperatures, which is a decisive factor in phenolic compounds extraction, as most of these processes are temperature dependent. In this work, the polyphenolic extracts obtained at 75 °C demonstrated high antioxidant activity. In another work, the extraction time to obtain phenolic compounds from *Pinus radiata* bark was reduced by 98.3% using MAE compared with the Soxhlet technique process, which required 3 h [112]. In addition, the extraction time was significantly reduced in a study performed by Chupin et al. [115] using MAE to extract tannins, flavonoids, and sugars compared with hot water-based extraction. The time was reduced to 3 min instead of 2 h. Moreover, they compared different bark particle sizes and conclude that small particle size (400 μm) improved the amount of extracts obtained. Therefore, MAE can be considered as a simple and rapid method to extract phenolic compounds from *Pinus* bark. The extraction of other valuable pine faction, such as oil or sugars, may also be improved [72].

#### 4.2.3. Supercritical Fluid Extraction

The SFE process enhances solvents’ behavior by working at pressures and temperatures near or above the critical point. Supercritical fluids exhibit different physicochemical properties which are advantageous in solvent extractions: they possess gas-like properties, such as diffusion, viscosity and surface tension, and liquid-like density and solvation power. This mixed behavior, as both liquid and gas, of the solvent in the supercritical region facilitates and enhances mass transfer [116]. Some solvents used for SFE are ethane, argon, methanol, water, and carbon dioxide, being this last one the most commonly used due to its non-toxicity, safety, easy removal from the extract, and low critical temperature (near room temperature, which is particularly important for termolabile compounds). However, for polar polyphenols extraction and owing to its non-polar character, CO_2_ is usually used in combination with co-solvents such as solvents as ethanol. In this way, the solvating power of CO_2_ is increased [117]. Typically, for polyphenols extraction, fractionated SFE is performed, where a first extraction is carried out usually with supercritical CO_2_ followed by a second extraction adding a polar solvent to increase the solubility of the phenolic compounds. For example, Braga et al. [118] performed a fractionated SFE of maritime pine bark; in the first extraction step, low-polarity CO_2_ soluble compounds were removed, and in the second step, they added 10% EtOH into the system to extract polar compounds and obtained a higher recovery yield of catechin and epicatechin than with Soxhlet extraction. Since the addition of EtOH enhances the extraction of polyphenols, Seabra and co-workers [119] studied the influence of the CO_2_:EtOH ratio and concluded that a 30:70 ratio was the most appropriate mixture to obtain the highest extract from maritime pine bark in the shortest time. An optimization study of SFE carried out by Ghoreishi et al. [120] achieved a 34% taxifolin recovery from *Pinus nigra* bark using ethanol as solvent at a flow rate of 1/20 of CO_2_. They also proposed a prediction model that can be used to scale up the SFE process for taxifolin extraction, thus reinforcing that SFE is a suitable process for polyphenols extraction. Therefore, SFE proved to have advantages over conventional extraction processes by reducing the amount of solvent used, being manageable in a way that specific compounds can be extracted and operating at low temperatures, which preserves the quality of the extracts. However, the final prices of products obtained with high-pressure technologies tend to be higher compared with conventional processes. 

#### 4.2.4. Pressurized Liquid Extraction

The PLE method is also referred to as accelerated solvent extraction, pressurized solvent extraction, and enhanced solvent extraction [36,106]. This technology is based on the use of liquid solvents at temperature and pressure values above the atmospheric boiling point and below the critical point values, decreasing the viscosity of the solvent, promoting accelerated dissolution kinetics, and increasing the solutes’ solubility. It also increases mass transfer rates and decreases surface tension, facilitating the penetration of solvents into the matrix, changing its structure, and disintegrating it [106,121]. For example, in the case of water as an extraction solvent in PLE (subcritical water extraction), the water is heated over 100 °C increasing pressure above atmospheric conditions. In the particular case of water, the dielectric constant decreases and water can reach behaviors similar to organic solvents [4,106,122]. Moreover, the possibility of using organic solvents decreases the polarity of the extraction fluid, making the extraction more selective and directed to the compounds of interest, such as phenolic compounds [122]. This method is considered a viable eco-friendly alternative to replace other extraction techniques, such as SFE or Soxhlet, which have the disadvantages of being expensive and, in the last case, of being slow and using large amounts of organic solvents.

In recent years, PLE has been widely applied in the extraction of antioxidant compounds from different plants, by-products, and agro-industrial waste [87]. Moreover, PLE has been used for recovering antioxidant phenolic compounds with industrial potential from different parts of pine plants [81,111,123]. Lixia et al. [81] investigated the influence of different extraction technologies in the antioxidant compounds of oils from *Pinus koraiensis* nuts. The results of this study concluded that subcritical extraction preserves the quality of pine nuts oil, containing considerable amounts of fatty acids, tocopherols, and tocotrienols. In addition, Liazid and co-workers [111] applied subcritical water technology at 100 °C and 4 Mpa conditions to obtain phenolic extracts from seeds of *Pinus maritima* and *Pinus d’Alpes*. In another work using pine leaves (*P. taiwanensis* and *P. morrisonicola* needles) as raw material [123], the combination of enzymes and ethanol using the PLE technique showed interesting results in obtaining bio-functional extracts with high amounts of phenolic compounds.

#### 4.2.5. Ohmic Heating Extraction

OH is a non-pulsed electrotechnology based on the conversion of electric energy into thermal energy with technological purposes [124]. This technique is based in the Joule effect (heat is generated inside a conductive matrix, in the presence of an electric current) and provides a fast and homogeneous heating rate in a semi-conductive material (0.1–10 S/cm). One advantage is the reduction of energy consumption in comparison to other heating extraction techniques [100,125]. The voltage applied in the OH process normally varies between 400 and 4000 V (electric field from 0.001 to 1 kV/cm), and the heating rates achieved depend on the power supply output, the equipment (reactor design), and the properties of the matrix (such as conductivity and viscosity) [124]. 

Furthermore, this “green” technology allows to reduce the ecological impacts caused by the extraction processes, decreasing the water use and waste generation [126]. OH can further induce not only thermal but also promote the formation of pores (electro-permeabilization) in cell membranes, showing a promising potential to obtain extracts that are more sterile [100,127]. This phenomenon is considered to be relevant in the extraction of bioactive compounds from different agri-food wastes and forestry by-products [125,128,129,130].

This novel and emergent electrotechnology has been proposed for the extractions of phenolic compounds from *Pinus pinaster* bark [59]. Interestingly, OH (5–15 V/cm) in combination with a hydroethanolic solvent (EtOH 50%) showed a marked increase in the extraction yield of antioxidant phenolic compounds compared to conventional solid–liquid extraction with the same solvent in a thermal bath (approximately 90 to 40 mg gallic acid equivalent /g bark, respectively). Specifically, the individual phenolic compounds such as taxifolin, quercetin, narigenin, apigenin, resveratrol, and some phenolic acids are most benefited by ohmic extraction. In this work, the effect of electric fields on the pine bark tissues disruption was visible, and a reduction of more than 50% in energy consumption compared to conventional heating was achieved.

Besides OH, pulsed electric fields (PEF) could also be an interesting option to explore, as it acts by destroying the cell wall and plant tissue structure and facilitating extraction. Though to our knowledge it has never been applied to pine matrices, it was successfully applied in the extraction of different compounds from many agri-food wastes (e.g., Norway spruce bark, tomato, and potato peels, among others) [131,132,133].

## 5. Polyphenols as Extracted Biocompounds

Polyphenols are chemical compounds distributed in herb plants, vegetables, and fruits with a wide range of applicability. Currently, more than 8000 phenolic compounds are known, and among them, 4000 flavonoids have been identified. The polyphenols are secondary metabolites essential for the growth and development of the plants [68]. They also protect the plants against insects and other animals. Polyphenols in plants are involved in functions related with sensory properties such as color, bitterness, and astringency.

**Figure 4:**
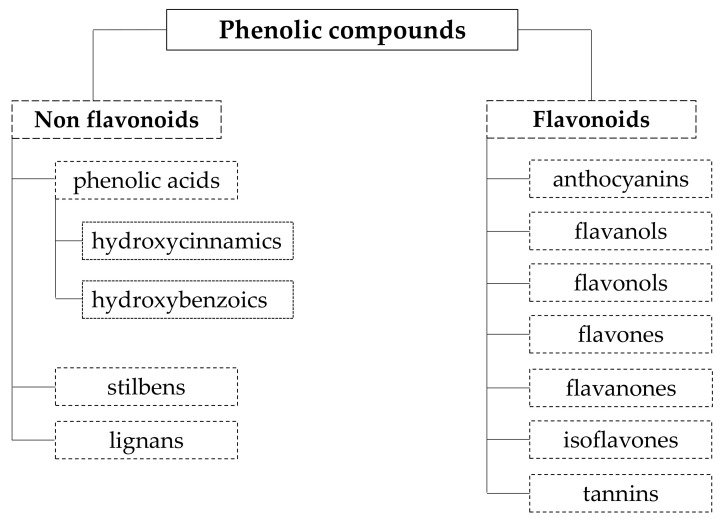
Polyphenol groups according to their chemical structure.

The common characteristics between all polyphenols are the presence of benzene ring(s) and hydroxyl groups. However, they are highly diverse and can be divided in several sub-groups. There are different ways of categorizing these compounds, based on their source of origin, biological function, or chemical structure. According to their chemical structure, polyphenols can be divided in two main groups: flavonoid and non-flavonoid. The non-flavonoid group incorporates the phenolic acids (hydroxybenzoic acids and hydroxycinnamic acids), stilbenes, and lignans. The flavonoids include compounds from the groups of anthocyanins, flavanols, flavonols, flavones, flavanones, isoflavones, and tannins (Figure 4). Phenolic acids are present in the free and bound form and can be divided in two main groups: hydroxybenzoic acids and hydroxycinnamic acids. Hydroxybenzoic acids are based on a C_6_–C_1_ structure and include protocatechuic, vanillic, gallic, and syringic acids. Hydroxycinnamic acids are compounds with a three-carbon side chain (C_6_–C_3_) and include coumaric, caffeic, and ferulic acids [135]. The demand of phenolic acids is very high in the industries as they work for precursors of other significant bioactive molecules, which are needed on regular basis for therapeutic, cosmetics, and food industries [136]. Phenolic acids are also available commercially as dietary supplements.

Stilbens are small number of compounds, composed of a 1,2-diphenylethylene nucleus with some hydroxyls. The main representative of this group is the resveratrol with strong antioxidant and anti-inflammatory properties. Lignans are produced by the oxidative dimerization of two phenylpropane units and are found in many plants, in particular in flax seeds [135].

The group of flavonoids is the most studied of both groups. Anthocyanins are polyphenols that determine the color of plant raw materials, imparting them a red, blue, purple, or pink color [137]. Tannins are highly polymerized substances and one of the most widespread organic compounds in nature. They have a relatively high molecular weight. Tannins can be further divided into two sub-groups, including hydrolysable tannins and condensed tannins [135]. The hydrolysable tannins are subdivided to gallotannins and ellagitannins, while condensed tannins are oligomers or polymers of flavan-3-ol monomers, which are linked by an interflavan carbon bond.

The distribution of the polyphenols in plants is not uniform. The phenolic content depends on factors such as the stage of ripening, time of harvest, and environmental factors [137]. In plants, the majority of polyphenols are linked with different sugar units at different positions of the polyphenol skeleton. 

Before extraction of the polyphenol, the raw material must be collected, properly transported, and stored. The handling of the sample before extraction is extremely important, as polyphenols are unstable molecules that can be easily oxidized. They can be easily deteriorated by light and high temperatures. Before storage, normally, samples are dried, frozen, or lyophilized. The extraction of phenolic compounds from plant materials depends on the nature of the sample matrix and also on the chemical properties of desired phenolic compounds, such as the number of aromatic rings and hydroxyl groups in its structure, polarity, and concentration [136].

The main solvents used for the extraction of these compounds are water, methanol, ethanol, acetonitrile, and acetone, or their mixtures with different proportions of water. Depending on the solvent used for the exaction, a mixture of phenolics soluble in the solvent will be extracted from plant materials. The choice of solvent used influences the phenolic final composition and bioactivity of the extract [59].

There is no universal extraction procedure suitable for the extraction of all plant phenolics [122]. For the estimation of total phenolics, flavonoids, and anthocyanins content, spectrophotometric methods are used [100,129]. Although these methods are rapid and simple, they do not give any information about individual compounds. To be identified and quantified, the phenolic compounds must be separated first. The most used method for polyphenol separation is the high-performance liquid chromatography (HPLC) coupled with a diode array detector (DAD). For the unequivocal identification, a mass spectrometric detector is usually used after the chromatograph (LC/MS). 

The search of functional products enriched with polyphenol extracts has been exploding. The aim is to increase the products’ antioxidant activity, giving rise to one or more types of biological activity [111]. Polyphenols are known to have diverse bioactivities. They are strong antioxidants, together with the vitamins and carotenoids, acting against the oxidative stress caused by reactive oxidative species. This is due to the hydroxyl group present in the molecule of the polyphenols. The hydrogen ion is dissociated and neutralizes the free radicals and other reactive oxygen species, scavenging the free radicals [138]. In that sense, the antioxidant power of a phenolic compound depends mostly on its chemical structure: for the phenolic acids, the number of hydroxyl groups in the molecule is the main driver [139]; in other polyphenols, the double bonds of the benzene ring and the double bond of the oxo functional group are also important [140].

There is controversy in the studies trying to connect the antioxidant capacity with the total phenolics content of a sample. In some studies, the total phenolic compounds were highly correlated with the antioxidant power of the samples [59]. In others, no significant correlations between total phenolic compounds and antioxidant power were found, such as in the case of wine samples [139]. Moreover, some groups of polyphenols were found to be more correlated to the antioxidant capacity of the extracts than others. It was concluded that the antioxidant activity has very strong correlation with anthocyanins and total procyanidins content. However, no correlation was found between the antioxidant capacity and the content of flavonols, flavanols (sum of (+)-catechins and (−)-epicatechins), and total gallotannins [137]. In addition, it was concluded that procyanidins and anthocyanins are included in the polyphenols responsible for the total antioxidant capacity of the investigated rhubarb varieties. Another study concluded that flavonoids and phenolic acids, as the main components of *R. maderensis*, are responsible for its antioxidant properties [141].

Moreover, extracts from medicinal plants in which the polyphenols are the main constituents are also known to have anti-diabetic properties [142]. For instance, *R. maderensis* extracts exhibited important inhibitory capacity toward key enzymes linked to type-2 diabetes and obesity [141]. 

Polyphenols have become target study compounds in the fight against cancer, as they are natural compounds that are safe and of low toxicity. Polyphenols are able to prevent cancer by reducing or blocking the harmful effects of free radicals on cells through their antioxidant properties [143]. Phenolic extracts of vine pruning residues demonstrated a decrease in the cell proliferation of four different cancer cell lines [144]. Polyphenols from oolong and black tea demonstrated various health benefits including anticancer, antioxidant, anti-cardiovascular, antimicrobial, anti-hyperglycemic, and anti-obesity activities [145]. Polyphenol extracts rich in β-carotene and rutin showed to have not only antioxidant but also anti-inflammatory effect [146]. Polyphenol extracts also demonstrated antimicrobial activity [144,147]. 

Lately, a lot of studies refer to the wood bark as an important source of polyphenols with a potential biological effect [68]. Extracts from pine contain a considerable amount of flavonoids and condensed tannins [115]. The amount of these active constituents varies depending on the pine specie and geographical location of growth [71]. Moreover, the solvent polarity and different methods of extraction used contribute to the different content in natural antioxidant and antioxidant activity of the extracts [88]. It is very important to ensure the chemical stability of polyphenols during the extraction processes, using mild extraction methods. The emergent technologies mentioned before in this review are important options to be considered [111].

As previously mentioned, different parts of the pine can be used for the extraction of polyphenols compounds (needles, seeds, bark, and cone), but the most studied is the pine bark. In Europe, there are 14 different known pine species. Although all extracts from pine have high amounts of total polyphenols regardless of the solvent, the method, the plant part or pine species used, there are differences in the concentrations and type of the individual compounds as well as in the strength of the bioactivities. This is due to the natural variability such as genotype, differences in growing and harvesting conditions, climate, soil type, etc. In a comparative study of three different species of pine bark extracts (*P. pinea*, *P. pinaster,* and *P. halepensis*), it was found that all extracts had induced cell-cycle arrest and apoptosis in Caco-2 cells (human colorectal adenocarcinoma). However, the extracts were different in terms of individual polyphenol compounds and the strength of the bioactivities. The extract of *P. pinaster* was the one with the highest biological activity and the one with the highest amount of procyanidin B2. The most abundant compounds in the pine samples were taxifolin and catechin. Procyanidin A2 was only present in samples of *P. halepensis.* Procyanidin B1 was found in *P. pinea* in concentrations two times higher compared to the other two species of pine in the study. Extracts of *P. pinaster* had the highest antioxidant capacity, while *P. halepensis* had the lowest antioxidant capacity [148].

Barks of various pine species from different regions of Turkey (*P. pinea*, *P. sylvestris*, *P. nigra*) and Germany (*P. parfliora*, *P. ponderosa*, *P. sylvestris*, *P. nigra*) were compared in terms of their flavonoids content and antioxidant activity [71]. The highest antioxidant activity was achieved by *P. pinea* (81.0%), while *P. parfliora* showed the lowest activity (31.9%). Moreover, a very good correlation was found between the antioxidant activity of the extracts and its total phenolic compounds, i.e., species *P. pinea* had the highest total phenol content, while *P. parfliora* had the lowest total phenol content. The main polyphenol compounds found in all extracts were catechin, catechin gallate, epicatechin, and taxifolin. The *P. pinea* had the highest amount of individual phenolic compounds.

Four different methods (maceration with magnetic stirring, ultrasound-assisted extraction, microwave-assisted extraction, and extraction with pressurized liquids) were used to obtain the extracts of seeds from *P. pinaster*. A direct relationship was found between the antioxidant activity and total polyphenol content of pine seeds extracts. Moreover, it was concluded that high extraction temperatures in any of the methods used brought lower bioactivities [111].

Needle and twig extracts of five different pine species (*P. brutia*, *P. halepensis*, *P. nigra*, *P. pinea*, and *P. sylvestris*) together with Pycnogenol^®^, a pine bark commercial extract, besides the strong antioxidant activity presented cholinesterase inhibitory potential [149]. Extract from *Pinus brutia* bark had 3.3-fold more total catechins and 9.8-fold more taxifolin than Pycnogenol^®^, showing strong anti-inflammatory activity [150]. A total of 17 phenolic compounds (mainly flavonoids) were identified in the needles of four pine species, *P. peuce*, *P. nigra*, *P. mugo,* and *P. sylvestris* from the Macedonian flora [113]. The authors concluded that there are no differences between the studied species of pine in terms of polyphenols. Taxifolin and quercetin were not found in any of the Macedonian pine species.

Moreover, the impact of the particle size of pine bark (between 0.05 and 1 mm) in the extracts was evaluated [115]. Mass transfer kinetics and the access of the solvent to the soluble components depends on the particle size [76]. The particle size has a direct effect on the amount of the polyphenol extracted. The smaller the particle size is, the more extracts are obtained (best results were registered for size 0.4 mm). However, there is no impact on the nature of the extract and on the types of the compounds extracted [115]. There is a lower limit of the particle size beyond which the quantity of extracted polyphenols decreased. It was registered that very fine particles stayed in suspension at the surface of the solvent and therefore were not subjected to proper extraction [76].

The extraction of polyphenols would also depend on the solid/liquid ratio. Meullemiestre et al. [76] found the optimum ratio to be about 6 g of dry material/100 mL; when concentrations were higher than 7.5 g of dry material/100 mL, the maritime pine wood absorbed all available liquid. 

Eighteen phenolic compounds were identified in the extracts of *P. pinaster* by Ferreira-Santos and co-workers [45,59]. In one of the studies, the authors tried to understand the action of the type of solvent (water and ethanol) and method of extraction (conventional or ohmic heating) over the chemical profile of the extracts. Extracts made with the different solvents were found to be statistically different in terms of content of phenolic compounds [59]. The antioxidant activity of the extracts were always higher in the hydroethanolic extracts comparing with the aqueous extracts. Moreover, significant correlations were found between total phenolic content and antioxidant activities of the obtained extracts [59]. In a second study, from the same author, pine bark extracts made with different concentrations of ethanol (from 0% to 90%) were evaluated for their bioactivities (antioxidant, antimicrobial, and antidiabetic) and in vitro cell viability (in normal and cancer cell lines). The study demonstrated that the pine bark extracts have high potential antioxidant, antidiabetic, and antimicrobial activities, especially when made with 50% and 70% of ethanol [45]. Moreover, the authors concluded that pine bark extracts act selectively on cancer cells, as these are negatively selected and the non-tumor cells are not.

In general, both studies [45,59] showed that the compounds with the highest concentrations in all samples were ellagic acid and taxifolin. The concentrations of ellagic acid accounted for between 9.0% and 50% of the total phenolic compounds, while taxifolin accounted for between 15% and 42% of the total phenolic compounds. Individual concentrations of phenolic compounds such as catechin, taxifolin, quercetin, caffeic acid, o-coumaric acid, ferulic acid, and ellagic acid in the extracts made with 50% ethanol were almost twice as high as in the correspondent extracts obtained with water. 

There are not many studies showing the identification and even less studies showing the quantification of polyphenols in extracts from *Pine species*. The extracts are obtained mostly from the pine bark, and fewer are obtained from needles (Table 3). The chemical composition of the extracts depends on the type of pine used (species, location), on the part of the plant, on the method of extraction, and on the solvent. For example, the main group of polyphenols compounds found in the *P. sylvestris* is the group of stilbenes [151], while in *P. pinaster*, the main group of compounds are the flavonoids [59].

As one can see from Table 3, the main polyphenol compounds found in extracts from pine needles are *p*-coumaric acid and epicatechin, in pine seeds eriodictyol and taxifolin, and in pine bark catechin, gallic acid, and taxifolin. In the following text, we summarize the bioactivities of the individual compound found in the extracts of pine species. However, we want to draw the attention of the reader to the idea that, in terms of expressing biological activities, the polyphenols act as group of compounds rather than individual compounds, and synergistic and/or antagonist or simply different effects may be found.

The *p*-coumaric acid together with ferulic and caffeic acids are the most common hydroxycinnamic acids in pine-based extracts. The hydroxylation of *p*-coumaric acid results in the formation of ferulic acid, while the oxymethylation of *p*-coumaric acid produces caffeic acid, respectively. These phenolic acids are used as precursors in the synthesis of lignins and other phenolics [136]. Taofiq et al. [156] conducted a study on individual compounds as possible ingredients in cosmeceutical formulations. The authors concluded that *p*-coumaric, protocatechuic, and cinnamic acids displayed anti-tyrosinase, antimicrobial, and anti-inflammatory activities, showing their potential for the cosmeceutical industry. Caffeic acid and, at a higher degree, ferulic acid proved to protect the skin against UVB-induced erythema. Besides as antioxidants, these two hydroxycinnamic acids can be used as photoprotectors in skin cosmetics [163].

The protocatechuic, vanillic, and syringic acids are the three commonly found hydroxybenzoic acids [136]. This tree hydroxybenzoic acids were also found in pine extracts (Table 3). Vanillic acid demonstrated anti-inflammatory activity with neuroprotective activity and was found to be a promising candidate for preventing and/or delaying the onset and progression of ischemic injury and vascular dementia [158]. In other cases, the use of a mixture of phenolic compounds rather than the individual compounds exhibits stronger activities. For example, the combined use of syringic acid, resveratrol, and gallic acid, in rats, revealed antioxidant and cardioprotective activities [159]. Resveratrol also showed an anticancer effect when examined in lung, prostate, breast, skin, and gastrointestinal cancers [172]. Resveratrol is the most known constituent of wines and grapes, but it was also found in pine bark extracts (Table 3). It was also proven that resveratrol has anti-inflammatory capacity, especially in the skeletal muscle, but it is less active in liver [171]. Moreover, gallic and *p*-coumaric acids were considered as promising adjuvant agents against the progression of neurodegeneration in the brain by diabetes [155]. Rosmarinic acid is known to have a number of potentially beneficial biological effects and is an acid ester of caffeic acid and 3(3,4-dihydroxyphenyl)lactic acid. The use of rosmarinic acid in gelatin edible film showed long-term antibacterial activity. Rosmarinic acid edible films may have promising application in the fields of food and pharmaceutical packaging, as they showed a good antibacterial activity even after 3 months of storage [173]. Rosmarinic acid was found in extracts of pine bark in concentrations between 0.4 and 0.8 mg/g [59]. This acid was found to be the predominant compound of *Salvia* species. Strong correlations between the rosmarinic acid contents and bioactivites of *Salvia* samples were established [165]. Moreover, this acid demonstrated potent antiviral properties [164]. 3,4 dihydroxybenzoic acid is universal in the Angiosperm plants, as it is constituent of lignin. It is a strong antioxidant, as well as a neuroprotective against Aβ-induced neuronal damage [168]. This acid can be used in formulations for phytonematode control, as it showed nematicidal activity against juveniles of *M. incognita* [174].

As we can see from the Table 3, in different pine extracts, many catechin compounds with diphenylpropane (C_6_–C_3_–C_6_–) skeletons were found. Epigallocatechin, epigallocatechin gallate, and epicatechin are the main constituents of the leaves of *Camellia sinensis* (the tea plant), while catechin gallate is a minor polyphenolic constituent in green tea: 1.28% (by weight) of the total catechin content in green tea [166]. These catechins are responsible for the astringent and bitter taste of the green tea [153]. Catechin is the main phenolic compound present in *P*. *pinaster* bark extract followed by epicatechin and epicatechin gallate [161]. Gallocatechin was found also in Norway spruce and confirmed to be a strong inhibitor of melanin biosynthesis; however, there is little information on the biological activities of this compound [162]. All these catechins have strong antioxidants and anticancer activities against different types of cancer [166]. Catechins have received considerable attention as promising candidates for development of therapeutic agents.

Taxifolin, as an individual compound, is extensively studied. It was found in the pine extracts of seeds and bark; in bark, it is present in much higher concentrations than in seeds [45,59,157]. For instance, this compound was recovered from *P*. *nigra* bark [120] with a maximum extraction recovery of 34%. Taxifolin was detected as the major compound in other needle leaved trees such as the Japanese larch, *Larix kaempferi* [175]. Its main bioactivities are antioxidant, anticancer, and anti-inflammatory [148,167]. Quercetin is a taxifolin-related flavonoid found in onions, and it showed anti-inflammatory, antimicrobial, and anticancer properties (in vitro and in vivo) [176]. Quercetin-rich extracts from onion skin can be used in functional bread production [176].

According to Lantto and co-workers [157], eriodictyol was one of the main compounds found in extracts of Siberian pine bark. Eriodictyol, as taxifolin, can be find in citrus fruits. It has showed antioxidant and anti-inflammatory activities. Recent findings indicated that eriodictyol might be a new preventative agent for psteoarthritis [177]. Another promising therapeutic agent for the treatment of osteoarthritis is ellagic acid [169]. It is found in high concentrations in the ethanolic extracts of *P. pinaster* [59], which is also a constituent in the fruit peel of berries and nuts [169].

Apigenin is found abundantly in herbs, fruits, and vegetables (peppermint, grape fruit, parsley). It has potent antioxidant, anti-inflammatory, and anticancer properties [170]. Gascon et al. [148] found three procyanidin compounds in pine bark extracts: A2, B1, and B2. The activities of procyanidins depend on their structure, especially on their degree of polymerization. Procyanidin B2 is one of the most active molecules within the procyanidins, as well as the most studied. It is also found in cocoa and grape seeds. The three compounds have antioxidant activity; B-type procyanidins have also neuroprotective activity. The richest set of bioactivities agglomerates for the procyanidins B2, but as we mentioned, this is also the most studied one.

There are several pine bark commercial extracts: Oligopin^®^, Pycnogenol^®^, and Flavangenol^®^. Pycnogenol^®^ is the most known and most studied one. Its extraction involves standardized consecutive steps using water and ethanol as solvents. It is a polyphenol-rich extract prepared from *P. pinaster* (French maritime pine). The main constituents are procyanidins (85%), flavonoids (catechin, taxifolin), as well as some phenolic acids in minor amounts (gallic, caffeic, and ferulic acid) [149]. This extract proved to have excellent antioxidant properties that can promote various health properties such as cardioprotective, anticancer, antihypertensive, and anti-inflammatory [118,150]. In another study, the clinical efficiency of Pycnogenol^®^ in the management, treatment, and control of chronic venous insufficiency and venous microangiopathy was proven [178]. This extract showed also anti-diabetic property, as the supplementation of Pycnogenol^®^ to conventional diabetes treatment lowered glucose levels and improved endothelial function [179]. Oligopin^®^ is another extract obtained from the pine tree *P. pinaster* from a specific location in France (Landes of Gascony). Its production includes two extraction steps and one purification step. This methodology ensure that the obtained extract has a specific and constant composition. The main compounds found in Oligopin^®^ are flavonoids (catechin and taxifolin) and acids (ferulic, gallic, caffeic, *p*-coumaric, and protocatechic) [180]. 

As a final remark, the individual polyphenol compounds found in the extracts of pine bark have diverse bioactivities that align with the mentioned previously bioactivities of the hole extracts such as antioxidant, anticancer, cardioprotective, antidiabetic, anti-inflammatory, etc. Pine extracts have active ingredients that are useful for the food industry as supplements or natural pigments, for food preservation and as active food packaging. In the cosmetic formulations, they can be used for protecting the skin against oxygen reactive species, formed by pollution, stress, or ultraviolet reaction. 

## 6. Conclusions and Future Perspectives

Pine residues and by-products are an important source of biocompounds with high industrial interest. They can be recovered using the biorefinery concept, thus contributing to the circular economy.

More environmentally friendly techniques have been explored to avoid the large amounts of (organic) solvents, energy consumption, and waste generation typical of conventional solvent extraction processes. Although replacing conventional technologies by non-conventional ones has emerged, improvements are necessary in terms of deep knowledge of the extraction processes and scaling up. UAE, MAE, SFE, PLE, and OH are some of these emerging promising technologies for bioactive compounds extraction in alignment with the Green Chemistry principles. Regarding the extraction step, the selection of the most appropriate techniques differs according to the type of compounds targeted for recovery and final aimed functionality/application. However, there is not a universal extraction method suitable for the extraction of all pine phenolics. The different pine species have different individual phenolic composition. For example, the main phenolic compound found in extracts of Portuguese pine is the taxifolin, whereas it was not found in Macedonian pine species. Depending on the final purpose for the extraction, an individual study must be carried on to tune the best extraction procedure.

Interestingly, all extracts from pine, regardless the solvent, the method, pine species, and the plant part used, have high amounts of polyphenols. 

However, there are differences in the concentrations and type of the individual compounds as well as in the strength of the bioactivities. There are not many studies showing identification and even less studies showing quantification of the individual polyphenols in extracts from pine species. 

Pine extracts have a number of described bioactivities that may be beneficial for the human health. As a consequence, pine extracts have high potential as constituents in formulation for the food, cosmeceutical, and pharmaceutical industries. 

The possible reutilization of the pine residues is yet limited, compared to its potential. In this context, more studies are needed to find and develop new products and uses resulting from pine residues and by-products.

## Figures and Tables

**Table 1:** *Pinus* trees species implemented in European countries, as well as its geographical distribution.

Latin Name	Common Name	Geographical Distribution
***Pinus sylvestris***	Scots pine	All countries of Europa and Asia
***Pinus nigra***	European black pine	Mountain areas of Europe, United States, and Asia Minor
***Pinus brutia***	Brutia pine	Eastern Coast of the Mediterranean (Turkey, Greece, Italy)
***Pinus pinaster***	Maritime pine	Western Mediterranean Sea, Central and Southern Europe, and North Africa
***Pinus halepensis***	Aleppo pine	Coastal areas of the Western Mediterranean region, Southern France and Italy, and North Africa
***Pinus cembra***	Swiss stone pine	Continental Alps and regions of the Carpathian Mountains
***Pinus uncinata***	Mountain pine	Mountains of Western Europe, Northern Europe, and Mediterranean
***Pinus pinea***	Stone pine	Mediterranean Basin, extending from Portugal to Syria
***Pinus strobus***	White pine	Eastern North America and Carpathian Mountains in Czech Republic and Southern Poland
***Pinus mugo***	Mountain pine	Mountains of Central and Eastern Europe
***Pinus heldreichii***	Bosnian pine	Southern and Western part of the Balkans, near the Mediterranean basin
***Pinuscontorta***	Lodgepole pine	Western North America, Europe, and New Zealand
***Pinus peuce***	Macedonian pine	Mountain areas of the Balkan Peninsula
***Pinus radiata***	Monterey pine	Central Coast of California, Australia, New Zealand, Mexico, Argentina, Chile, Uruguay, Kenya, Spain, and South Africa

**Table 2:** Green technologies for phenolic compounds recovery from pine by-products and possible applications.

Extraction Method	Pine Species	Part of Tree	Optimum Extraction Conditions	Foreseen Applications	Reference
**Ultrasound-Assisted Extraction** **(UAE)**	*P. pinaster* *P. d’Alpes*	seeds	Water; 75 °C; 20 min	Bioactive extracts for food supplements	[111]
	*P. radiata*	bark	Acetone (70%, *v/v*); 25 °C; 6 min; 35 kHz/85 W	Nutraceutical action	[112]
	*P. mugo* *P. nigra* *P. peuce* *P. sylvestris*	needles	Methanol (70%, *v/v*); RT; 30 min	Medicinal and pharmaceutical	[113]
	*P. pinaster*	wood	Acidified water; 40 °C; 43 min; 0.67 W/cm^2^	Diet supplement	[76]
**Microwave-Assisted Extraction (MAE)**	*P. pinaster* *P. d’Alpes*	seeds	Water; 75 ºC; 20 min	Bioactive extracts for food supplements	[111]
	*P. radiata*	bark	Acetone (70%, *v/v*); 25 °C; 1–2 min; 2450 MHz/900 W	Bioactive extracts as promising pharmaceutical and food applications	[112]
	*P. pinaster*	bark	Ethanol (80%, *v/v*); 3 min; 100W	Diet supplement	[115]
	*P. pinaster*	bark	92.4 min; 803.5 W	Antioxidant essential oils	[72]
	*P. koraiensis*	cones	Enzymatic pretreatment; ionic liquid–lithium salt; 15.95 min; 581.49 W	Cosmetic or health-related applications	[99]
**Pressurised Liquid Extraction** **(PLE)**	*P. taiwanensis* *P. morrisonicola*	needles	Enzymatic hydrolysis (ethanol 70% *v/v*); 70 °C; 180 min; 4.12 MPa	Food, cosmetic, or health applications	[123]
	*P. pinaster* *P. d’Alpes*	seeds	Water; 100 °C; 20–23 min; 4 MPa	Bioactive extracts for food supplements	[111]
	*P. koraiensis*	seeds	*n*-butane; 21 °C; 0.5 MPa	Oils for cosmetology and pharmaceutical application	[81]
**Supercritical Fluid Extraction** **(SFE)**	*P. pinaster*	bark	CO_2_ + ethanol (10%, *v/v*); 50 °C; 35 min; 25 MPa	Aroma/flavor, food, and pharmaceutical industries	[118]
	*P. pinaster*	bark	CO_2_:ethanol (30:70, *v/v*); 30 °C; 360 min; 25 MPa	Antioxidant extracts	[119]
	*P. pinea* *P. sylvestris* *P. nigra* *P. parviflora* *P. ponderosa*	bark	CO_2_ + ethanol (3% *v/v*); 40 and 60 °C; 200 bar	Food, cosmetic, or health-related applications	[71]
	*P. niruri*	--	CO_2_; 90 min; 60 °C; 30 MPa	Antioxidant extracts	[88]
	*P. nigra*	bark	CO_2_ + ethanol; 42.8 °C; 137.9 min; 19.3 MPa	Pharmaceutical industry	[120]
	*P. pinaster*	wood	CO_2_ + ethanol (10%, *v/v*); 50 °C; 35 min; 25 MPa	Antioxidant extracts for food and pharmaceutical applications	[134]
**Ohmic Heating Extraction** **(OH)**	*P. pinaster*	bark	Ethanol (50%, *v/v*); 83 °C; 30 min; 5-15 V/cm	Antioxidant extracts for food and pharmaceutical applications	[59]

**Table 3:** Individual phenolic compounds found in pine by-products and their reported bioactivities.

Name	Chemical Formula	Concentration Range (mg/g)	Bioactivities	Reference
**NEEDLES**				
**Epicatechin**	C_15_H_14_O_6_	1.5	antioxidant	[152,153]
***p*-Coumaric Acid**	C_9_H_8_O_3_	2.3	antioxidant, anti-inflammatory, hepatoprotective and renoprotective, anti-neurodegenerative, anti-cholesterolemic, improve insulin resistance, anti-tyrosinase, antimicrobial	[152,154,155,156]
**SEEDS**				
**Protocatechuic Acid**	C_7_H_6_O_4_	0.5	anti-tyrosinase, antimicrobial, and anti-inflammatory activities	[156,157]
**Catechin**	C_15_H_14_O_6_	0.5	hepatoprotective activity	[145,157]
**Epigallocatechin Gallate**	C_22_H_18_O_11_	0.5	antimicrobial, antioxidant, photoprotective	[145,157]
**Vanillic Acid**	C_8_H_8_O_4_	0.9	anti-inflammatory, neuroprotective	[157,158]
**Syringic Acid**	C_9_H_10_O_5_	1.0	cardioprotective, antioxidant, antimicrobial, anti-inflammatory, neuro and hepatoprotective activities	[157,159]
**Epicatechin**	C_15_H_14_O_6_	1.3	antioxidant	[157]
**Taxifolin**	C_15_H_14_O_7_	1.7	antioxidant, anticancer, anti-inflammatory	[157]
**Cinnamic Acid**	C_9_H_8_O_2_	0.1	anti-tyrosinase, antimicrobial, and anti-inflammatory	[156,157]
**Eriodictyol**	C_15_H_12_O_6_	3.8	anti-inflammatory	[157,160]
***m*-Coumaric Acid**	C_9_H_8_O_3_	traces	not found	[157]
**BARK**				
**Gallic Acid**	C_7_H_6_O_5_	traces–5.5	anti-inflammatory, antihyperlipidemic, antioxidant, antitumor, antihyperglycemic, and anti-neurodegenerative, cardioprotective	[59,154,155,159,161]
**Gallocatechin**	C_15_H_14_O_7_	0.07–0.95	inhibitor of melanin biosynthesis	[45,59,162]
**Epicatechin**	C_15_H_14_O_6_	0.06–1.9	antioxidant	[71,161]
**Epicatechin Gallate**	C_22_H_18_O_10_	0.3–0.9	antioxidant	[161]
**Catechin**	C_15_H_14_O_6_	0.095–7.7	antioxidant, anticancer, cardioprotective, antifungal	[45,59,71,161,162]
**Vanillic Acid**	C_8_H_8_O_4_	0.02–0.07	neuroprotective, anti-inflammatory	[45,59,158]
**Caffeic Acid**	C_9_H_8_O_4_	0.03–0.2	antioxidant, photoprotective	[45,59,163]
**Rosmaniric Acid**	C_18_H_16_O_8_	0.4–0.8	antioxidant, antidiabetic, antibacterial, antiviral	[59,164,165]
**Catechin Gallate**	C_22_H_18_O_10_	0.002–1.5	antioxidant, anticancer	[71,166]
**Taxifolin**	C_15_H_12_O_7_	0.01–4.7	antioxidant, anticancer, anti-inflammatory	[45,59,148,167]
**3,4 Dihydroxy-Benzoic Acid**	C_9_H_10_O_4_	0.08–0.8	neuroprotective, antioxidant, nematicidal activity	[45,59,168]
**Ellagic acid**	C_14_H_6_O_8_	0.4–4.0	anti-inflammatory, antioxidant	[45,59,169]
**Naringin**	C_27_H_32_O_17_	0.8–2.0	not found	[45,59]
**Apigenin**	C_15_H_10_O_5_	0.3–0.5	anticancer, antioxidant, anti-inflammatory	[45,59,170]
**Resveratrol**	C_14_H_12_O_3_	0.03–0.4	antioxidant, anti-cancer, cardioprotective, anti-inflammatory	[45,59,159,171]
**Ferulic acid**	C_10_H_10_O_4_	0.06–0.5	antioxidant, photoprotective	[45,59,163]
***p*-coumaric acid**	C_9_H_8_O_3_	n.q.	antioxidant, anti-inflammatory, hepatoprotective and renoprotective, anti-neurodegenerative, anti-cholesterolemic, improve insulin resistance, anti-tyrosinase, antimicrobial	[59,154,155,156]
**Quercetin**	C_15_H_10_O_7_	0.06–1.1	inflammatory, antimicrobial, anticancer	[45,59]
**Procyanidin A2**	C_30_H_24_O_12_	n.q.	antioxidant	[148]
**Procyanidin B1**	C_30_H_26_O_12_	n.q.	antioxidant, neuroprotective, anti-proliferative activity	[148]
**Procyanidin B2**	C_30_H_26_O_12_	n.q.	antioxidant, anti-inflammatory, cardioprotective, neuroprotective, anti-proliferative activity	[148]

n.q.–not quantified.
